# A new bacterial tRNA enhances antibiotic production in *Streptomyces* by circumventing inefficient wobble base-pairing

**DOI:** 10.1093/nar/gkac502

**Published:** 2022-06-14

**Authors:** Ximing Chen, Shuyan Li, Binglin Zhang, Haili Sun, Jinxiu Wang, Wei Zhang, Wenbo Meng, Tuo Chen, Paul Dyson, Guangxiu Liu

**Affiliations:** Key Laboratory of Desert and Desertification, Northwest Institute of Eco-Environment and Resources, Chinese Academy of Sciences, Lanzhou, Gansu, China; Key Laboratory of Extreme Environmental Microbial Resources and Engineering, Lanzhou, Gansu, China; School of Medical Information and Engineering, Xuzhou Medical University, Jiangsu, China; Key Laboratory of Extreme Environmental Microbial Resources and Engineering, Lanzhou, Gansu, China; State Key Laboratory of Cryospheric Sciences, Northwest Institute of Eco-Environment and Resources, Chinese Academy of Sciences, Lanzhou, Gansu, China; School of Chemistry and Environmental Science, Lanzhou City University, Lanzhou, Gansu, China; Key Laboratory of Desert and Desertification, Northwest Institute of Eco-Environment and Resources, Chinese Academy of Sciences, Lanzhou, Gansu, China; Key Laboratory of Extreme Environmental Microbial Resources and Engineering, Lanzhou, Gansu, China; Key Laboratory of Desert and Desertification, Northwest Institute of Eco-Environment and Resources, Chinese Academy of Sciences, Lanzhou, Gansu, China; Key Laboratory of Extreme Environmental Microbial Resources and Engineering, Lanzhou, Gansu, China; Key Laboratory of Biological Therapy and Regenerative Medicine Transformation Gansu Province; The First Clinical Medical School of Lanzhou University, China; Key Laboratory of Extreme Environmental Microbial Resources and Engineering, Lanzhou, Gansu, China; State Key Laboratory of Cryospheric Sciences, Northwest Institute of Eco-Environment and Resources, Chinese Academy of Sciences, Lanzhou, Gansu, China; Institute of Life Science, Swansea University Medical School, Singleton Park, Swansea SA2 8PP, UK; Key Laboratory of Desert and Desertification, Northwest Institute of Eco-Environment and Resources, Chinese Academy of Sciences, Lanzhou, Gansu, China; Key Laboratory of Extreme Environmental Microbial Resources and Engineering, Lanzhou, Gansu, China

## Abstract

We report the discovery and functional characterization of a new bacterial tRNA species. The tRNA-Asp-AUC, from a fast-growing desert streptomycete, decodes GAU codons. In the absence of queuosine tRNA anticodon modification in streptomycetes, the new tRNA circumvents inefficient wobble base-pairing during translation. The tRNA, which is constitutively expressed, greatly enhances synthesis of 4 different antibiotics in the model mesophilic species *Streptomyces coelicolor*, including the product of a so-called cryptic pathway, and increases yields of medically-important antibiotics in other species. This can be rationalised due to increased expression of both pleiotropic and pathway-specific transcriptional activators of antibiotic biosynthesis whose genes generally possess one or more GAT codons; the frequency of this codon in these gene sets is significantly higher than the average for streptomycete genes. In addition, the tRNA enhances production of cobalamin, a precursor of S-adenosyl methionine, itself an essential cofactor for synthesis of many antibiotics. The results establish a new paradigm of inefficient wobble base-pairing involving GAU codons as an evolved strategy to regulate gene expression and, in particular, antibiotic biosynthesis. Circumventing this by expression of the new cognate tRNA offers a generic strategy to increase antibiotic yields and to expand the repertoire of much-needed new bioactive metabolites produced by these valuable bacteria.

## INTRODUCTION

In addition to the threat of viral pandemics, the World Health Organization recognizes that ‘antibiotic resistance is one of the biggest threats to global health, food security, and development today’ (https://www.who.int/news-room/fact-sheets/detail/antibiotic-resistance). One mitigation is to speed up discovery and production of new antibiotics. Approximately 70% of our antibiotics in current usage are derived from natural products produced by *Streptomyces* bacteria (1). These gram-positive non-motile filamentous bacteria undergo elaborate morphological and physiological differentiation (2). In favourable conditions, a spore will germinate with the hyphal germ tube extending by apical growth and occasional branching to form a mycelial network that penetrates a localised nutrient source. Subsequently, the bacteria erect aerial hyphae that grow away from the nutrient source with the uppermost regions of these aerial hyphae undergoing regular coordinated cell division to generate chains of new spores. Often concomitant with aerial growth, many different primary metabolites are converted into complex bioactive molecules, such as antibiotics, by metabolic pathways encoded by specialized biosynthetic gene clusters (BGCs). A typical streptomycete genome contains upwards of 20 different BGCs specifying secondary metabolites, but typically only a small fraction of these are expressed in laboratory conditions at levels permitting isolation and structural determination of their products (3). The remaining BGCs that are poorly expressed or silent specify what are termed cryptic or orphan pathways. An example of a cryptic pathway is that for coelimycin in the model streptomycete *S. coelicolor* A3(2); obtaining sufficient yields of this antibiotic was only achieved by first deleting a gene encoding a pathway-specific transcriptional repressor (4), or by both deleting other BGCs that compete for precursors and incorporating mutations in the *rpoB* gene encoding the ß-subunit of RNA polymerase (5). A simpler generic strategy to increase BGC expression, including those of cryptic pathways, would be fundamental to new antibiotic discovery programmes.

The universal genetic code is degenerate in that it has 61 codons for 20 amino acids and three stop codons. However, the number of distinct tRNA genes present in a genome is less, meaning that some tRNAs recognize more than one codon, as initially explained by Crick's Wobble Hypothesis (6). The Wobble Hypothesis assumes that the first two base-pairs between the tRNA anticodon and the mRNA codon are canonical (Watson-Crick) base pairings, but that the third base-pair can involve non-canonical base pairing. The Modified Wobble Hypothesis, proposed in 1991, introduced the concept of tRNA modifications that contribute to define the specificity of wobble base-pairing at the third position (7). Essentially, while some tRNA base modifications promote wobble base-pairing by impacting the structure of the tRNA anticodon loop, other types of modification reduce the possibility for wobble base-pairing. As modifications can customize the decoding capacity of tRNAs, they potentially have an important role in regulating gene expression (8). An example of the importance of anticodon modification concerns how aspartate codons are decoded in bacteria. There are two different aspartate codons, GAU and GAC, and bacteria typically decode both with a single tRNA-Asp-GUC. Queuosine (Q) is a tRNA modification that occurs in the wobble anticodon position of tRNAs with GUN anticodon sequences (9) to favour increased translational efficiency. Q can pair with U as well as C bases located at the respective position of the corresponding mRNA codons and in this way a single modified bacterial tRNA species can efficiently decode both aspartate codons.

Notable features of typical sequenced genomes of free-living mesophilic *Streptomyces* include their high G + C content (> 70 %) and a large linear chromosome (typically > 8 Mb), consisting of a ∼6.5 Mb ‘core’ genome and variable sized left and right arms (10–12). Genome annotation has revealed a large investment in contingency functions, particularly in the chromosome arms, with evidence of genes acquired by horizontal transfer, enabling these bacteria to survive the challenges of a variable terrestrial environment. Of interest is whether streptomycetes adapted to live in extreme environments have significantly different genomes and whether they produce different types of antibiotics. To address this, we sequenced the small, 6.5 Mb genome of a fast-growing *S. violaceusniger* strain SPC6 (hereafter termed SPC6), isolated from the Linze Desert of North West China (13). Here we describe the function of a novel tRNA-Asp-AUC found in the SPC6 genome. We demonstrate how its heterologous expression both in the model streptomycete, *S. coelicolor*, and in a range of *Streptomyces* species used by the pharmaceutical industry, promotes precocious and increased production of antibiotics, including coelimycin in the former. The results reveal a new paradigm for the regulation of gene expression based on the efficiency of translation of GAT codons and how this impacts antibiotic biosynthesis.

## MATERIALS AND METHODS

### Bacterial strains and plasmids

*Streptomyces* species and strains used in this study are listed in Table 1. *Escherichia coli* JM109 (14) was used as an intermediary for cloning, and *E. coli* ET12567 (15) was used as a donor for intergeneric conjugation of plasmids into *Streptomyces*. The fate of the transferred plasmids, which lack streptomycete plasmid replication functions, is to integrate into the chromosomal phiC31 *attB* site by site-specific recombination (16). To construct modified eGFP genes possessing between 0 and 6 GAT codons transcribed from the ermE* promoter, the promoter and 5′ end of the gene sequences were used to design two overlapping complementary primers for each example.

**Table 1.** Streptomyces species

*Streptomyces*	Reference
*S. violaceusniger* SPC6	12
*S. coelicolor* M145	14
*S. chattanoogensis* L10	27
*S. clavuligerus* ATCC 27064	28
*S. filamentosus* NRRL 11379	29
*S. peucetius* ATCC 29050	30

0GAT:

- Primer 0F (BamHI): ATGGATCCCTGTTGTGGGCACAATCGTGCCGGTTGG
- Primer 0R (NdeI): ATCATATGCATCGCTGGATCCTACCAACCGGC

1GAT:

- Primer 1F (BamHI): ATGGATCCCTGTTGTGGGCACAATCGTGCCGGTTGG
- Primer 1R (NdeI): ATCATATGATCCATCGCTGGATCCTACCAACCGGC

2GAT:

- Primer 2F (BamHI): ATGGATCCCTGTTGTGGGCACAATCGTGCCGGTTGGTAGG
- Primer 2R (NdeI): ATCATATGATCATCCATCGCTGGATCCTACCAACCGGCACG

4GAT:

- Primer 4F (BamHI): ATGGATCCCTGTTGTGGGCACAATCGTGCCGGTTGGTAGG
- Primer 4R (NdeI): ATCATATGATCATCATCATCCATCGCTGGATCCTACCAACCGGCACGATTGTGC

6GAT:

- Primer 6F (BamHI): ATGGATCCCTGTTGTGGGCACAATCGTGCCGGTTGGTAGGATCCAGCGATGG
- Primer 6R (NdeI): ATCATATGATCATCATCATCATCATCCATCGCTGGATCCTACCAACCGGCACG

After annealing and DNA synthesis, the resulting fragments and the vector pIJ8660 (17) were digested with BamHI and NdeI and ligated together. The resulting plasmids pIJRCG-0, pIJRCG-1, pIJRCG-2, pIJRCG-4, pIJRCG-6 were verified by sequencing.

The tRNA-Asp-AUC and upstream sequences of SPC6 were amplified using the primers pt1: CGCGTGACGCTCGTCTCCAGG and pt2: CGATCGCCGGGCAGCGTACG. The 562 bp amplicon and the vector pIJRCG-6 and pSH152 (18), previously digested with the EcoRV, were ligated together to create, respectively pIJTCG and pSHTCG. To create a mutated copy of the tRNA-Asp-AUC, primers P1: AAAGATATCACAGGGTCACCGCCGGCTCCCC and P2: CGACGTCCTCCAGCTTGAcGCGTGGGCGCGAC were used to generate a 170 bp amplicon. In parallel, primers P3: GTCGCGCCCACGCgTCAAGCTGGAGGACGTCG and P4: AATCTAGAGGGGGAAGGGATCGGCTGCGTC were used to generate a 150 bp amplicon. Fusion PCR, with primers P1 and P4, were then used to generate the fused 282 bp product that was digested with EcoRV and XbaI. This was cloned into the EcoRV and XbaI sites of pSH152. All plasmids were verified by sequencing.

### Growth conditions

*Escherichia coli* strains were grown on LB medium (19). Intergeneric conjugation of plasmids into *Streptomyces* strains was carried out on MS medium supplemented with 10 mM MgCl_2_ (15).

Proteomes were analysed after growth on nutrient agar (Oxoid) or a starch-based minimal medium (Gauze's synthetic medium no. 1). Media for fermentations of producers of commercial antibiotics are described in the section ‘Antibiotic analyses’, below.

### RNA purification, sequencing and qRT-PCR

RNA was extracted from 0.1 g (wet weight) of mycelia with TRIzol Reagent (Invitrogen) following the manufacturer's protocol. The RNA was subsequently ethanol precipitated and resuspended in DEPC-treated water. RNA was quantified using a Nanodrop and the quality of the RNA sample was assessed using a 2100 Bioanalyzer. A protocol for analysing inosine modifications in tRNA was followed (20), by first preparing RNA libraries with an NEB Next Multiplex Small RNA Library Prep Set for Illumina following the manufacturer's protocol. Samples were loaded in a 3% agarose gel and fragments between 160 and 220 bp were selected. Sequencing was performed in a HiSeq2000 SR100 Illumina sequencer running four samples per lane. For qRT-PCR, cDNAs were obtained from 1 μg of total RNA using a PrimeScript RT reagent Kit (Takara); using the manufacturers recommendations with random decamers in a reaction volume of 20 μl. cDNAs were diluted 1/15 in nuclease free water. qRT-PCR was carried out on 1 μl of diluted cDNA with a Power SYBR® Green Master Mix (ThermoFisher). Gene specific primers used for quantitative PCR were designed using Beacon Designer 7.8 (Premier Biosoft, USA). The following primers were used,

- for tRNA-Asp-AUC,
- forward: AAGGCTGTAGCGCAGAGGT, reverse: AGACCGGTCGGATTCGAAC
- for tRNA-Asp-GUC,
- forward: CGGTCCTGACGGGATTTGA, reverse: CCTGTGGAGCAGTTTGGAGT
- for *hrdB*, used as internal control to normalise samples,
- forward: CCACTCAGTGGAAGAACGTACT, reverse: TTCGCTGCGACGCTCTTTCG.

The specificity of each reaction was assessed using melt curve analysis. Relative transcript abundance was determined using a comparative CT method, comparing tRNA Ct values with those for *hrdB*.

### eGFP expression analysis

For fluorescence microscopy, strains were grown on the starch-based minimal medium (Gauze's synthetic medium no. 1) in the angle under a coverslip inserted obliquely into the agar (16). To quantify expression, *S. coelicolor* strains were grown up on the same starch-based minimal medium for 3 days at 30°C. 0.1 g mycelium (cell wet weight) was suspended in 10 ml phosphate buffer and hyphae lysed with an ultrasonicator. Fluorescence due to eGFP expression in lysates was determined using a fluorescence microplate reader (excitation 488 nm, emission 509 nm).

### Proteome analyses

iTRAQ proteomic technology, coupled with liquid chromatography with tandem mass spectrometry (LC-MS/MS), was used to analyze proteins quantitatively and identify those that were differentially abundant (21). iTRAQ analysis in our study was performed by Shanghai Lu-Ming Biotech co., Ltd., Shanghai, China.

Bacterial cells pellets were suspended in cooled acetone (1 h, −20°C), centrifuged (15 000 × g, 15 min, 4°C), and dried with a vacuum freeze dryer. The samples were resuspended in cold saturated-phenol (pH 7.5) and shaken (30 mins, 4°C). The upper phenolic phase was collected by centrifugation (5000 × g, 30 min, 4°C), 5 volumes of cold 0.1 M ammonium acetate in methanol was added, and then it was stored (1 h, −20°C). After centrifugation (5000 × g, 30 min, 4°C), the pellets were washed and mixed with 2 volumes of ice-cold methanol. The pellets were centrifuged, dried and dissolved in lysis solution (1 h, 30°C). The supernatants were isolated by centrifugation (15 000 × g, 15 min). Protein concentrations were measured with the BCA method (22), after which they were stored at −80°C for iTRAQ analyses. Additionally, 10 μg samples were subjected to 12% SDS-PAGE, visualized and then scanned according to Candiano's protocol (23).

The filter-aided sample preparation (FASP) method (24) was adopted for enzymatic hydrolysis of the proteins (100 μg). After 50 μl trypsin (50 ng/μl) digestion, peptides were labeled according to the manufacture's protocol for 8-plex iTRAQ reagent (AB SCIEX, USA).

The dried samples were resuspended with 100 μl buffer A, after which reversed-phase liquid chromatography (RPLC) was employed on an Agilent 1200 HPLC System (Agilent). The first segment was collected from 0 to 5 min, after which each additional segment was collected at a 4.5 min interval for 6–45 min, while the last segment was collected from 46 to 50 min for a total of 10 segments. Each segment was dried and used for subsequent RPLC–MSMS analyses.

In brief, samples were resuspended with Nano-RPLC buffer, filtered through a C18 nanoLC trap column, and a Chromxp C18 column (75 μm × 15 cm, C18, 3 μm 120 Å). The Eksigent nanoLC-UltraTM 2D System (AB SCIEX) was used to perform the online Nano-RPLC. Triple TOF 5600 system (AB SCIEX, USA) was used to analyze MS data combined with Nanospray III source (AB SCIEX, USA).

Data were processed with the Protein Pilot Software v. 5. 0 (AB SCIEX, USA) against the NCBI database using the Paragon algorithm (25). The results of protein quantification were obtained by the matching of tandem mass spectrometry (MS) data and theoretical data, and was performed with the search option: emphasis on biological modifications.

An Orbitrap Elite high-resolution mass spectrometer (Thermo Fisher Scientific, USA) was used for ITRAQ quantitative proteomic analyses. Normalized high-energy collision dissociation (HCD) was performed, with the collision energy set at 30%. A protein database search and quantification were performed using Maxquant 1.5.1.0 (Thermo Fisher Scientific, USA). Oxidation (M) and acetyl (protein N-term) were used as the variable modifications and carbamidomethyl (C) was the fixed modification. The MS/MS tol. (FTMS) was 20 ppm.

### Antibiotic analyses

Antibiotic assays were conducted on samples from three biological replicates. Actinorhodin and undecylprodiginine production was quantified according to standard protocols (16,26). Production of the calcium-dependent antibiotic was assayed using *Bacillus mycoides* as an indicator strain (27). Coelimycin biosynthesis was indicated by zones of inhibition against *B. mycoides* in the absence of supplementation with Ca^2+^, and the amount of the yellow-pigmented coelimycin P2 confirmed by spectroscopy (*A*_460_) of culture supernatants (4).

For the producers of commercial antibiotics, each species was grown in a medium optimised for production of the respective antibiotic:

- *S. chattanoogensis* L10: yeast extract 3 g/l, malt extract 3 g/l, tryptone 5 g/l, glucose 40 g/l (28);
- *S. clavuligerus* ATCC 27064: glycerol 15 g/l, soya peptone 10 g/l, malt extract 10 g/l, yeast extract 1.0 g/l, K_2_HPO_4_ 2.5 g/l, MgSO_4_.7H_2_O 0.75 g/l, MnCl_2_.4H_2_O, 0.001 g/l, FeSO_4_.7H_2_O, 0.001 g/l and ZnSO_4_.7H_2_O 0.001 g/l (29);
- *S. filamentosus* NRRL 11379: tryptone 17g/l, soya peptone 3g/l, glucose 2.5g/l, NaCl 5 g/l, K_2_HPO_4_ 2.5g/l (30);
- *S. peucetius* ATCC 29050: maltose 22.5 g/l, Difco yeast extract 5 .04 g/l, NaNO_3_ 4.28 g/l, K_2_HPO_4_ 0.23 g/l, HEPES 4.77 g/l, MgSO_4_·7H_2_O 0.12 g/l, NaOH 0.4 g/l and 2 ml/l of trace element solution containing: ZnCl_2_ 40 mg/l, FeCl_3_·6H_2_O 200 mg/l, CuCl_2_·2H_2_O 10 mg/l, MnCl_2_·4H_2_O 10 mg/l, Na_2_B_4_O_7_·10H_2_O 10 mg/l and (NH_4_) 6Mo_7_O_2_·4H_2_O 10 mg/l (31).

The different *Streptomyces* strains were first grown in 1 l of the respective medium in shake flasks for 7 days. Subsequently, 500 ml of mycelium was combined with 5 l of medium in a 10 l fermenter. Fermentations were carried out at 28°C, 200 rpm, 1 vvm, pH 6.8 (pH adjusted by addition of either ammonia or phosphoric acid).

To quantify yields, extracts were analysed using an HPLC HP1100 system equipped with a quaternary pump, a degassing device, an autosampler, a column thermostatting system, a diode-array detector (DAD), and Agilent Chem-Station for LC and LC/MS systems software. The observed peaks were compared with peaks obtained after chromatography of standards prepared for each respective antibiotic (all obtained from Shanghai Macklin Biochemical Company, Ltd).

To analyse natamycin biosynthesis, 2 ml of fermentation broth was added to the 25ml methanol, and the mycelia then lysed using ultrasound. After centrifugation at 12 000 rpm, supernatants were analysed by HPLC using a Kromasil ODS (C18) (150 × 3.20 mm i.d., 5 mm particle size) column maintained at 25°C. Acetonitrile (A) and Milli-Q water (B) were used as mobile phases. Samples were eluted in gradient mode. Three selected wavelengths were set in DAD detector, 291.4, 304.4 and 319.4 nm, corresponding to the three absorption peaks of the characteristic natamycin spectrum.

For daunorubicin, the mycelium in 10 ml of fermentation broth was lysed by ultrasonification, and the pH then adjusted to 5.0 by addition of HCl. The samples were then centrifuged at 12 000 rpm, and supernatants applied to a C18 reverse-phase octadecyl column. The mobile phase was 65% methanol and 35% phosphorylated water, pH 2.0. HPLC was set at a flow rate of 1 ml min^−1^ and *A*_254_ nm was measured.

To determine yields of clavulanic acid, the mycelium in 10 ml of fermentation broth was lysed using ultrasound. The pH was then adjusted to 8.0 by addition of KOH. Supernatants were obtained after centrifugation at 12 000 rpm and applied to a C-18 reversed-phase column after prederivatization with imidazole solution. The eluent was composed of 100 mM KH_2_PO_4_ at pH 3.2 with a buffer-to-methanol ratio of 94:6 and the flow rate was 2.5 ml min^−1^. The derivatives were detected at 311 nm.

For analysis of daptomycin, 10 ml of fermentation broth was combined with 10 ml methanol, and the mycelium lysed by ultrasound. Supernatants were obtained after centrifugation at 12 000 rpm and applied to a C-18 reversed-phase column. The mobile phase consisted of 0.1% trifluoroacetic acid in distilled water and acetonitrile (55:45, v/v). The flow rate was 1.0 ml/min and the wavelength for detection was 210 nm.

### Quantification of cobalamin

The strains with or without the tRNA were grown in the starch-based minimal medium for 3 days at 30 °C. 0.1 g mycelium (cell wet weight) was resuspended in 1 ml 50% methanol and lysed using ultrasound. HPLC analysis was performed on an Agilent 1260 Infinity system equipped with a 1260 quaternary pump and a 1260 diode-array detector. Chromatographic separation was carried out on an Ascentis Express C8 column (2.7 μm, 3 × 100 mm) with an Ascentis Express Guard column (2.7 μm, 3 × 5 mm) (Sigma-Aldrich, USA). The column was maintained at a temperature of 30°C and the sample volume injected was 10 μl. Optimal chromatographic separation was achieved at a flow-rate of 0.4 ml min^−1^ using a gradient with the mobile phase A (20 mmol l^−1^ potassium phosphate monobasic, in doubly distilled water, adjusted to pH 3 with phosphoric acid) and mobile phase B (HPLC-grade acetonitrile) as follows: 5–50% B in 8 min; then to 80% B in 0.1 min and held for 3.5 min; then back to 5% B in 0.1 min. The column was re-equilibrated for 3.3 min at the initial conditions (5% B). Each run was completed within 15 min. Cobalamin was detected at 228 nm.

### Bioinformatics

Genome annotation of the SPC genome employed RAST (Rapid Annotation using Subsystem Technology) (http://rast.nmpdr.org) (32). Related tRNA sequences were found using MEGABLAST (33), searching nucleotide databases at NCBI. Codon frequencies were calculated by dividing the number of a given codon by the size of the gene in base pair. tRNA secondary structure and Cove scores were determined using tRNAscan-SE 1.21 (34,35).

### Statistical analyses

All measurements were made on a minimum of three biological replicates. For pairwise statistical analyses of significance of fluorescence values and metabolite yields/activities, a Student's *t*-test was used. For analysis of proteome data, SPSS version 20.0 was used to analyze data by one-way ANOVA and Bonferroni's correction was performed to adjust for multiple comparisons.

## RESULTS

### Structure, function and genome context of a new bacterial tRNA-Asp-AUC

**Figure 1.**
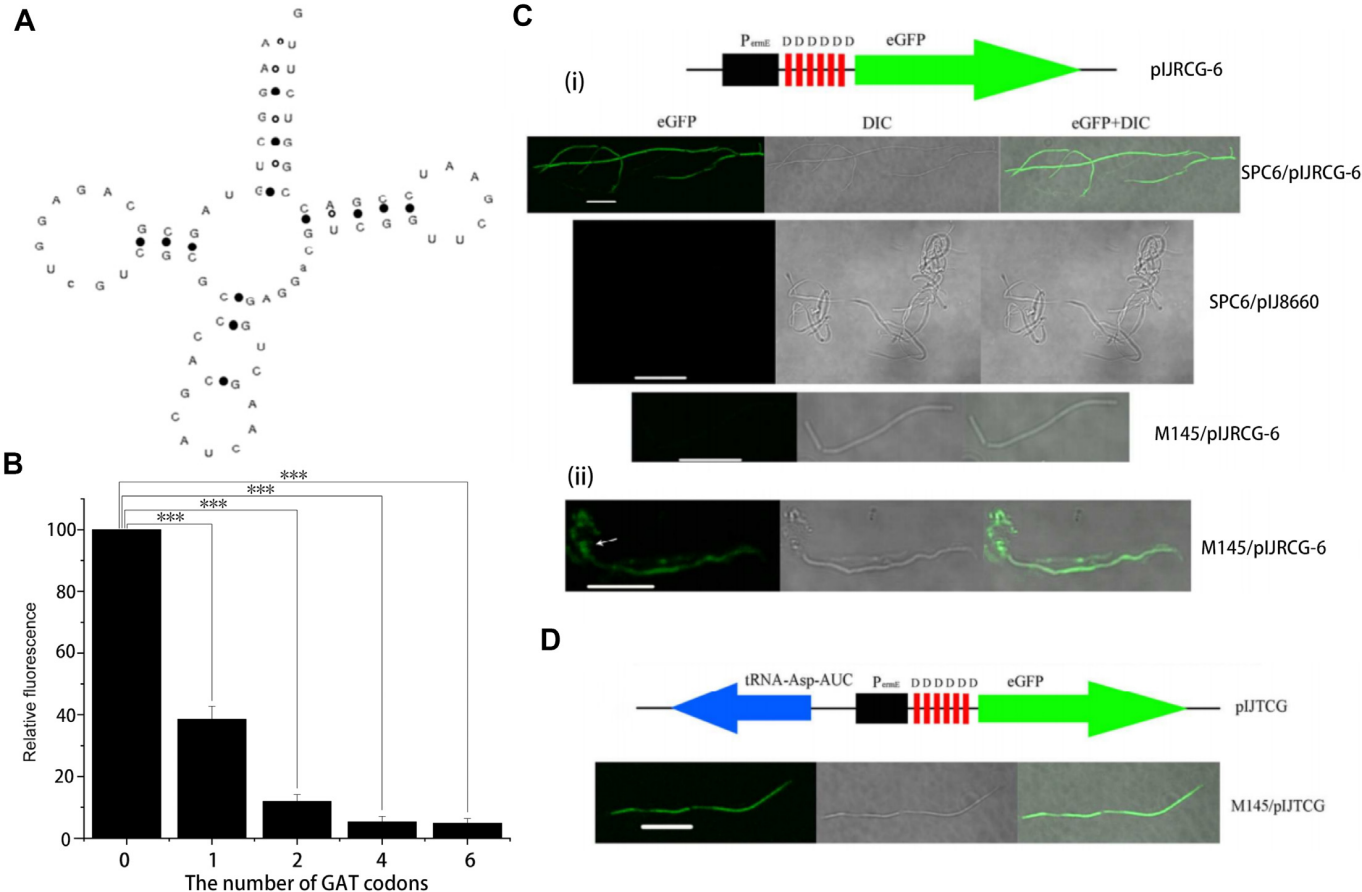
Structure and functional analysis of tRNA-Asp-AUC. (**A**) Predicted structure of the SPC6 tRNA-Asp-AUC. Solid circles represent G:C base pairs and open circles represent A/G:U base-pairs. (**B**) Relative eGFP expression in vegetative hyphae of *S. coelicolor* strains, each containing an integrated eGFP gene with from 0 up to 6 copies of an N-terminal GAT codon (D). Error bars indicate SD, *** signifies *P* ≤ 0.001. (**C**) (i) Fluorescence microscopy, differential interference contrast and merged images of vegetative hyphae of SPC6 and *S. coelicolor* M145 containing the integrated plasmid pIJRCG encoding a modified eGFP with 6 N-terminal aspartic acid residues translated from repeated GAU codons [D], as indicated above the hyphal images. Also shown are hyphae of SPC6 containing the cloning vector pIJ8660 with a promoterless unmodified copy of eGFP. Strains were grown for 3 days at their optimal temperatures (30°C for *S. coelicolor* M145 and 37°C for SPC6). (ii) Microscopy of a representative aerial hypha of *S. coelicolor* M145 containing pIJRCG, sampled after 6 days growth. The arrow indicates a spore. Bar = 10 uM. (**D**) Microscopy of a representative vegetative hypha, sampled after 3 days growth, of *S. coelicolor* M145 containing the integrated plasmid pIJTCG, in which the SPC6 tRNA-Asp-AUC is co-expressed, as indicated above the hyphal images. Bar = 10 uM.

Analysis of the tRNA complement of SPC6 revealed 75 tRNA genes encoding 44 distinct species. This complement includes a possible tRNA-Asp with an anticodon sequence AUC. This tRNA-Asp-AUC has a Cove score, a predictor of secondary structure stability and consequently function, of 29.08 with the stem of the anticodon loop stabilised by 3 G:C base-pairs (Figure 1A). Bacteria generally do not encode a tRNA-Asp-AUC. We searched 3 tRNA databases, GtRNAdb (http://gtrnadb.ucsc.edu/), tRNADB-CE (http://trna.ie.niigata-u.ac.jp/), and tRNAdb (http://trnadb.bioinf.uni-leipzig.de/), revealing a potential tRNA-Asp-AUC in just five bacterial genomes: *Rhodospirillum photometricum* DSM122, *Sphingomonas wittichii* RW1, *Streptomyces hygroscopicus* subsp. jingangenis, *Hymenobacter* sp. DG5B and *Bacillus thuringiensis* C15. We are not aware that the function of any of these potential tRNA species has been analysed to date and they do not share significant homology with the SPC6 tRNA-Asp-AUC.

A BLAST search for related bacterial tRNAs revealed 32 streptomycete genomes encoding an homologous potential tRNA-Asp-AUC (Supplementary Table S1), out of a total of 4649 sequenced streptomycete genomes (census date 12 August 2021), but no homologs in other sequenced organisms. In each of the 32 genomes, that includes the fully annotated genome of *S. collinus* Tu365, the tRNA gene is located within one or other chromosome arm. These regions typically encode non-essential contingency functions and often include genomic islands and BGCs (36). Scrutiny of the surrounding genes revealed a conserved cluster of 4 genes on the 3′ side of the tRNA encoding, respectively, a gala leucine-rich protein, a PE-PGRS protein related to those expressed by *Mycobacteria* (37), a MoxR-like ATPase and a conserved hypothetical protein. The frequency of the GAT codon in these genes does not differ significantly from the genome average for streptomycetes. Genes distal to these are not conserved between genomes, nor are genes to the other side of the tRNA gene. This suggests that the conserved gene cluster may be derived from an ancestral integrative element: many actinobacterial integrative elements are known to integrate at the 3′ end of tRNA genes (38).

To assess whether the tRNA could be modified to inosine at the first anticodon position, nucleotide 34, the tRNA was sequenced (small RNAseq) from both SPC6 and after its expression in a heterologous model streptomycete, *S. coelicolor*, that normally lacks a tRNA-Asp-AUC. Sequencing of the tRNA from both species revealed in both cases adenine with a frequency of 97% at position 34 in the anti-codon loop, and guanine at a frequency of 0.9% (Table 2). No other modifications in the tRNA sequence were detected using this method. Inosine is structurally a guanine analogue and, consequently inosines in nucleic acids are read as ‘G’ upon sequencing (39). The very low frequency of G at position 34 indicates that deamination of adenosine in the tRNA at this position is negligible and that the tRNA’s likely function is to decode GAT codons. We speculate that specific sequence features such as the purine at position 32 and the cytosine in place of uracil at position 33 may result in inefficient recognition of the tRNA by adenosine deaminase.

**Table 2.** Sequencing of tRNA-Asp-AUC

Base at position 34	Number of sequence reads	Percentage
**tRNA-Asp-AUC from SPC6**		
A	47 197	97.2%
T	432	0.89%
G	452	0.93%
C	476	0.98%
total number of sequences	48 557	100%
**tRNA-Asp-AUC from M145/pIJTCG**		
A	45 797	96.7%
T	516	1.09%
G	436	0.92%
C	611	1.29%
total number of sequences	47 360	100%

Given the unusual structure of the predicted tRNA-Asp-AUC, especially in relation to its anticodon stem, we needed to confirm its decoding functionality. We constructed modified eGFP genes with between 1 and 6 cognate GAT codons at the beginning of the open reading frame, expressed from the strong constitutive ermE* promoter (40), and introduced them into *S. coelicolor* lacking a tRNA-Asp-AUC. During vegetative growth, the amount of detectable fluorescence due to eGFP expression depended on the number of GAT codons present at the 5′ end of the gene (Figure 1C). The gene with a single GAT codon was expressed at approximately 40% of the level of the unaltered gene with no 5′ GAT codon. With 4 or 6 GAT codons, the level of expression was reduced to between 5 and 10% of the unaltered gene. The gene with 6 GAT codons was also introduced into SPC6. Fluorescence microscopy detected little or no fluorescent protein in vegetative hyphae of the recombinant *S. coelicolor* strain, whereas the highly fluorescent vegetative hyphae of the recombinant SPC6 indicated a high level of translation of the 6 repeated GAU codons (Figure 1B), that continued throughout the growth cycle. Accumulation of eGFP was observed only in older aerial hyphae of the recombinant *S. coelicolor* strain (Figure 1B). In this genetic background, translation of the mRNA is dependent on either wobble base-pairing by the sole Asp tRNA encoded by this species, tRNA-Asp-GUC (*S. coelicolor* encodes two identical copies of this tRNA), or mistranslation, although the latter is less likely given the repetitive codons within the mRNA. In addition, we expressed the modified eGFP gene with 6 GAT codons in an *S. coelicolor* strain also expressing the tRNA-Asp-AUC with its native promoter integrated into the chromosome. Fluorescent vegetative hyphae, indicative of efficient translation of the eGFP mRNA during the early growth phase, were observed in this strain (Figure 1D). This confirmed that, in the absence of the cognate tRNA, translation of GAU codons dependent on wobble base-pairing is inefficient during vegetative growth. Moreover, these experiments confirmed that the predicted tRNA-Asp-AUC does indeed efficiently decode GAT codons.

Efficient translation of the reporter gene with 6 GAT codons during early growth suggested that the tRNA-Asp-AUC is itself expressed early. We determined the abundance of both tRNA-Asp-AUC and tRNA-Asp-GUC in *S. coelicolor* at different time points, indicating constitutive expression of both tRNA species at equivalent levels to each other (Supplementary Table S2).

### tRNA-Asp-AUC impacts gene expression in the heterologous model streptomycete, *S. coelicolor*

Expression of the tRNA could be expected to affect translation of genes with GAT codons in a heterologous host such as *S. coelicolor*. *In silico* analysis of codon usage in this model species indicates that in the 7826 annotated genes of *S. coelicolor* M145 there are a total of 155 copies of the rarest TTA codon, known to limit translation of several key developmental genes (41), and, at the other extreme, 202 387 copies of the most abundant GCC codon (Supplementary Table S3). The overall pattern is that the most prevalent codons contain at least two guanine or cytosine bases, reflecting the high GC content of the typical streptomycete genome. Consequently, for the two aspartate codons, there is a total of 149 917 GAC codons, but only 7471 alternative GAT codons. These GAT codons are not evenly distributed, with approximately 3000 genes having a higher frequency than the genome average of 0.00096 GAT codons per bp, up to the highest frequency of 0.0179 GAT codons per bp (SCO0107). Some 4008 genes (51%) have no GAT codon. For comparison, we determined the frequency of GAT codons in the annotated genome of the kirromycin producer *S. collinus* that encodes an identical tRNA-Asp-AUC to that of SPC6. Interestingly, for the chromosome the frequency is not higher than that of *S. coelicolor* with 0.00087 GAT codons per bp, whereas the two large linear plasmids SCO1 and SCO2 (42) have much higher frequencies of 0.0026 and 0.0022 GAT codons per bp, respectively.

We predicted that expression of the tRNA-Asp-AUC could lead to more efficient translation of proteins encoded by genes with GAT codons. Indeed, a comparison of the proteomes of *S. coelicolor* strains with and without tRNA and harvested after growth on nutrient agar, at two time-points, 3 and 5 days, revealed substantial differences (Supplementary Table S4). As a consequence of expression of the tRNA, we documented overexpression of 213 proteins at day 3 and an overlapping set of 184 proteins overexpressed at day 5. In general, these overexpressed proteins contained a two-times higher frequency of the GAT codon in their genes (0.0015 per bp) compared to genes encoding the proteins that were not overexpressed (0.0007 per bp). The frequency of the GAT codon in the genes of overexpressed proteins was also higher than the average of 0.00096 per bp for *S. coelicolor* genes. Despite this trend favouring expression of genes with higher than average GAT codon content, the codon was absent from many highly expressed genes, suggesting that their expression may be dependent on regulatory pathways whose components are encoded by genes that may have GAT codons. Indeed, among the overexpressed proteins at day 3, several were transcriptional regulators with one or more GAT codons (e.g. SCO2015, SCO223, SCO2517, SCO4628, SCO4677, SCO6286 and SCO7146). Of note, in terms of biological function, were proteins encoded by two antibiotic BGCs. 8 and 5 proteins encoded by the undecylprodiginine (Red) BGC were overexpressed at days 3 and 5, respectively. In addition, six proteins encoded by the coelimycin (Cpk) BGC were overexpressed at both time points, despite overexpression of the pathway repressor ScbR encoded by SCO6265 that has 3 GAT codons (0.0046 per bp). ScbR-mediated repression is relieved by binding of the small molecule inducer gamma butyrolactone (43), so it is possible that this metabolite is also abundant in the given growth conditions. The *sbcA* (SCO6266) gene responsible for synthesis of the inducer contains two GAT codons (0.0021 per bp). ScbR also activates expression of the Act and CDA BGCs (44).

Growth conditions can influence the developmental phenotype and antibiotic production (45). To compare how growth conditions could influence the profile of proteins overexpressed because of the activity of the novel tRNA, the proteomes of cultures grown on a minimal medium were analysed after 3 days (Supplementary Table S5). In these conditions, 209 proteins were upregulated due to the tRNA, and of these only 27 were common with the upregulated proteins observed after 3 days growth on nutrient agar. Moreover, the average frequency of the GAT codon in the 209 genes was lower, at 0.0007 per base pair, indicating a less direct impact of the tRNA on gene expression by, for example, increasing translation of GAU-containing mRNAs of transcriptional regulators, translation factors and proteins impacting protein stability (e.g. SCO7134, SCO1758 and SCO4296). Of note, was overexpression of seven cobalamin biosynthesis proteins encoded by the gene cluster SCO1847 – SCO1857 (in specific *cobD, cobQ, cobN, cobB, cobI*, *cbhGH*). The average frequency of the GAT codon in this BGC is 0.0011 per bp (Supplementary Table S6). In addition, six proteins involved in biosynthesis of undecylprodiginine were overexpressed. The respective genes (*redR*, *redP, redO, redN, redL* and *redK*) are components of two operons that belong to the undecylprodiginine BGC. Two of these genes, *redR* and *redL*, were overexpressed due to the tRNA when the culture was grown on nutrient agar.

### The novel tRNA promotes precocious antibiotic production in *S. coelicolor*

**Figure 2.**
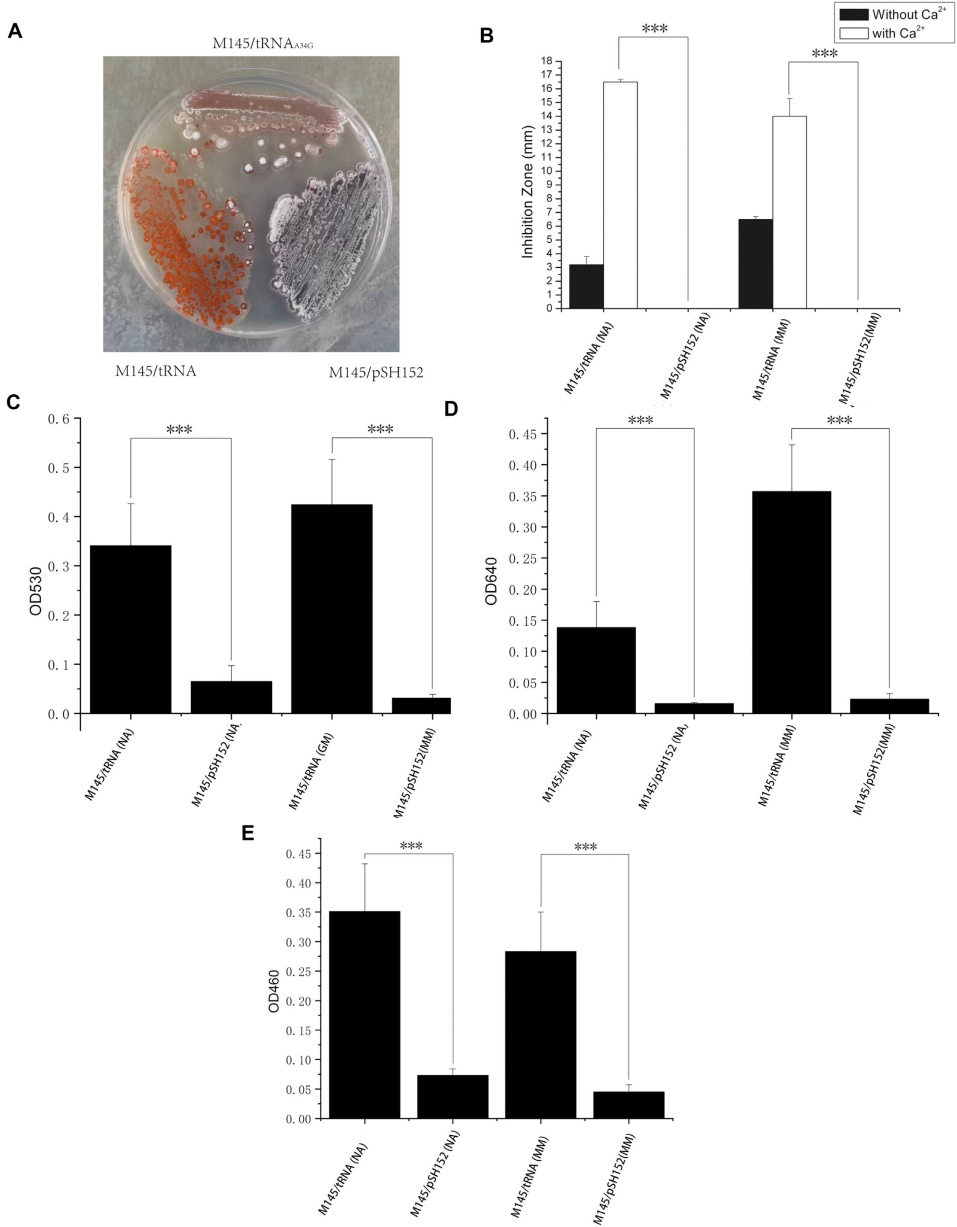
Enhanced production of antibiotics by *S. coelicolor* expressing tRNA-Asp-AUC. (**A**) Strains of *S. coelicolor* M145 containing the empty vector pSH152 (M145), pSGTCG (tRNA) and ptRNA_A34G_ were grown on minimal medium for 10 days. For (B–E), strains of *S. coelicolor* M145 containing the empty vector pSH152 (M145) or pSGTCG (tRNA) were grown on nutrient or minimal medium for 5 days prior to assays. Error bars indicate SD, *** signifies *P* ≤ 0.001. (**B**) A bioassay to determine inhibition of *Bacillus mycoides*. Zones of inhibition were determined after addition of *B. mycoides* in soft nutrient agar overlays with or without supplementation with calcium ions; (**C**) undecylprodiginine biosynthesis was determined by spectrophotometric assays (*A*_530_) of acidified methanol extracts; (**D**) actinorhodin biosynthesis was determined by spectrophotometric assays (*A*_640_) of alkalised culture supernatants; (**E**) yellow-pigmented coelimycin P2 was quantified by spectrophotometric assays (*A*_460_) of culture supernatants.

Based on the proteome analysis we expected a qualitative increase in synthesis of the undecylprodiginine by *S. coelicolor* expressing the tRNA-Asp-AUC. Indeed, after 10 days growth on minimal medium agar, this strain visibly produced more red-pigmented antibiotic compared to the control strain (Figure 2A), but was largely deficient in development of aerial hyphae and spore chains. We also mutated the A at position 34 in the anticodon loop to G, essentially converting the tRNA-Asp-AUC into another tRNA-Asp-GUC. Qualitatively, much less undecylprodiginine was produced when this mutated tRNA was expressed in *S. coelicolor* (Figure 2A), and development of aerial hyphae was largely restored.

To investigate quantitatively the overall impact of the tRNA on antibiotic biosynthesis, the two strains of *S. coelicolor*, with and without tRNA-Asp-AUC, were grown on the two different growth media used for analysis of the proteomes reported above. Initially, we assayed production of three different antibiotics: the benzoisochromanequinone dimer polyketide antibiotic actinorhodin, undecylprodiginines, and the calcium-dependent lipopeptide antibiotic (CDA). Precocious production of actinorhodin and the undecylprodiginines due to expression of the tRNA were observed during growth on both media (Figure 2C and D). A bioassay to detect CDA also indicated abundant synthesis of this antibiotic during growth on both media types, dependent on expression of the tRNA (Figure 2B). In control bioassays in which calcium nitrate was omitted in the soft nutrient agar overlay it was apparent that dimensions of zones of antibiotic activity were in part due to another antibiotic that could be coelimycin, previously termed a cryptic polyketide (Figure 2B). Consequently, we quantified the abundance of the yellow-pigmented coelimycin P2 in extracts, indicating that after 5 days growth on both nutrient agar and minimal medium there was substantial overproduction of this metabolite due to the tRNA (Figure 2E). Of particular note was the ability of the tRNA to promote synthesis of inhibitory amounts of both CDA and coelimycin in both growth conditions that do not support synthesis of inhibitory levels these compounds in the isogenic strain lacking the tRNA.

To assess if precocious synthesis of the different antibiotics could be attributed to the tRNA acting directly on expression of genes in the respective BGCs, the frequency of the GAT codon in each BGC was analysed (Supplementary Table S7). This revealed that only the actinorhodin BGC has a significantly higher overall GAT codon frequency of 0.0018 per bp compared to the genome average. However, each BGC contains at least one gene with a GAT frequency greater or equal to 0.0041 per bp. Increased expression of these genes dependent on the tRNA may increase antibiotic yields if the corresponding protein performs a rate-limiting biosynthetic step or has a regulatory activating function on expression of other genes in the BGC. Indeed, genes encoding pathway specific activators from all four BGCs contain at least one GAT codon (Supplementary Table S7). In addition, a fifth antibiotic BGC for methylenomycin biosynthesis is encoded on the plasmid SCP1 in the wild-type *S. coelicolor* A3(2). The methylenomycin pathway specific activator gene, *mmyB*, contains 6 GAT codons (0.0064 per bp).

Given that expression of the tRNA can result in overexpression of the pleiotropic regulator ScbR, we also considered other pleiotropic regulators of antibiotic BGCs in *S. coelicolor*. We examined the frequency of the GAT codon in a comprehensive list of 41 known pleiotropic activator genes in the *S. coelicolor* genome (46). 29 of these regulator genes contain at least one GAT codon and the overall frequency of the codon in this gene set is 0.0013, 1.5 times greater than the genome average (Supplementary Table S8).

### Increased yields of medically important antibiotics in different species expressing the tRNA

**Figure 3.**
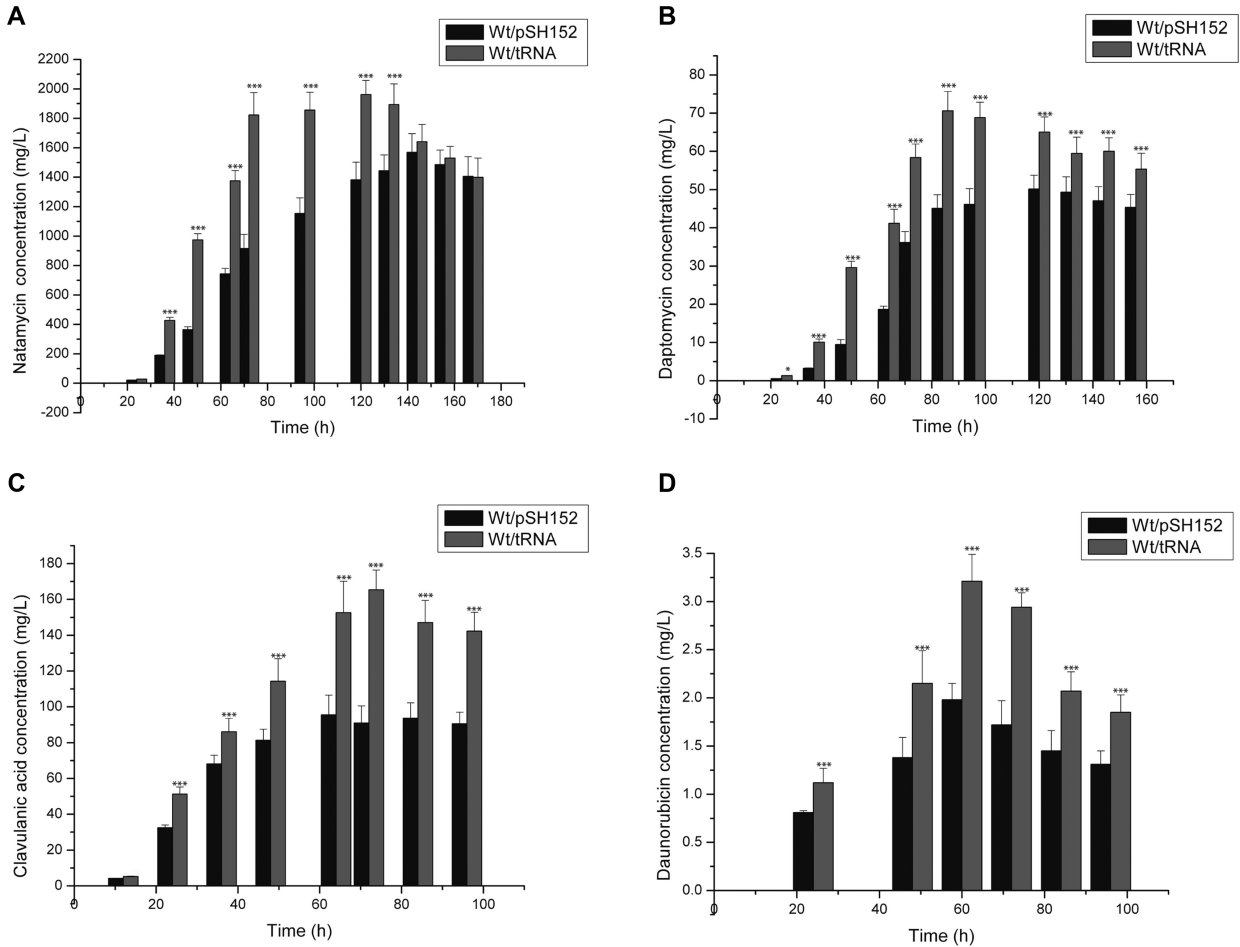
Expression of tRNA-Asp-AUC enhances biosynthesis of commercial antibiotics. Isogenic pairs of strains of producers of commercial antibiotics, with (pSGTCG) and without (pSH152) the expressed tRNA-Asp-AUC, were grown in a fermenter and production of the relevant antibiotic was determined at different time-points. Black bars indicate yields from the strain containing the empty vector and grey bars indicate yields from the strain expressing tRNA-Asp-AUC. (**A**) production of natamycin by *S. chattanoogensis*; (**B**) production of daptomycin by *S. filamentosus*; (**C**) production of clavulanic acid by *S. clavuligerus* and (**D**) production of daunorubicin by *S. peucetius*. Error bars indicate SD, * signifies 0.01 < *P* ≤ 0.05, *** signifies *P* ≤ 0.001 at the different time points.

To assess whether expression of the tRNA could be exploited as a generic means to improve antibiotic yields, the gene was transferred into 4 different species that normally lack the tRNA and that produce antibiotics used in medicine: *S. chattanoogensis*, a producer of the tetraene polyene antibiotic natamycin (47); *S. filamentosus* (also named *S. roseosporus*), a producer of the lipopeptide antibiotic daptomycin (48); *S. clavuligerus*, a producer of the β-lactam clavulanic acid (49); and *S. peucetius*, a producer of the polyketide anti-cancer drug daunorubicin (50). In each case, we compared antibiotic yields between the corresponding strains containing the empty vector and the cloned tRNA gene, grown in a 10 l fermenter in media optimised for production of the given antibiotic. For each antibiotic, we observed enhanced yields dependent on expression of the tRNA (Figure 3).

Analysis of codon usage revealed a higher frequency of the GAT codon in the complete daunorubicin and daptomycin BGCs compared to the genome averages for the respective species (Supplementary Table S9). In addition, the pathway-specific regulatory genes contained within all the BGCs contain a relatively high number of GAT codons compared to the respective genome averages for each species. We extended this analysis to examine GAT codons in pathway-specific regulatory genes from a wide range of streptomycete BGCs (Supplementary Table S10). All contain at least one GAT codon, with up to 9 in the *narR4* regulator (0.0096/bp) in the nanchangmycin BGC of *S. nangchangensis*, indicating that the tRNA would favour expression of pathway specific regulators in all species examined.

**Figure 4.**
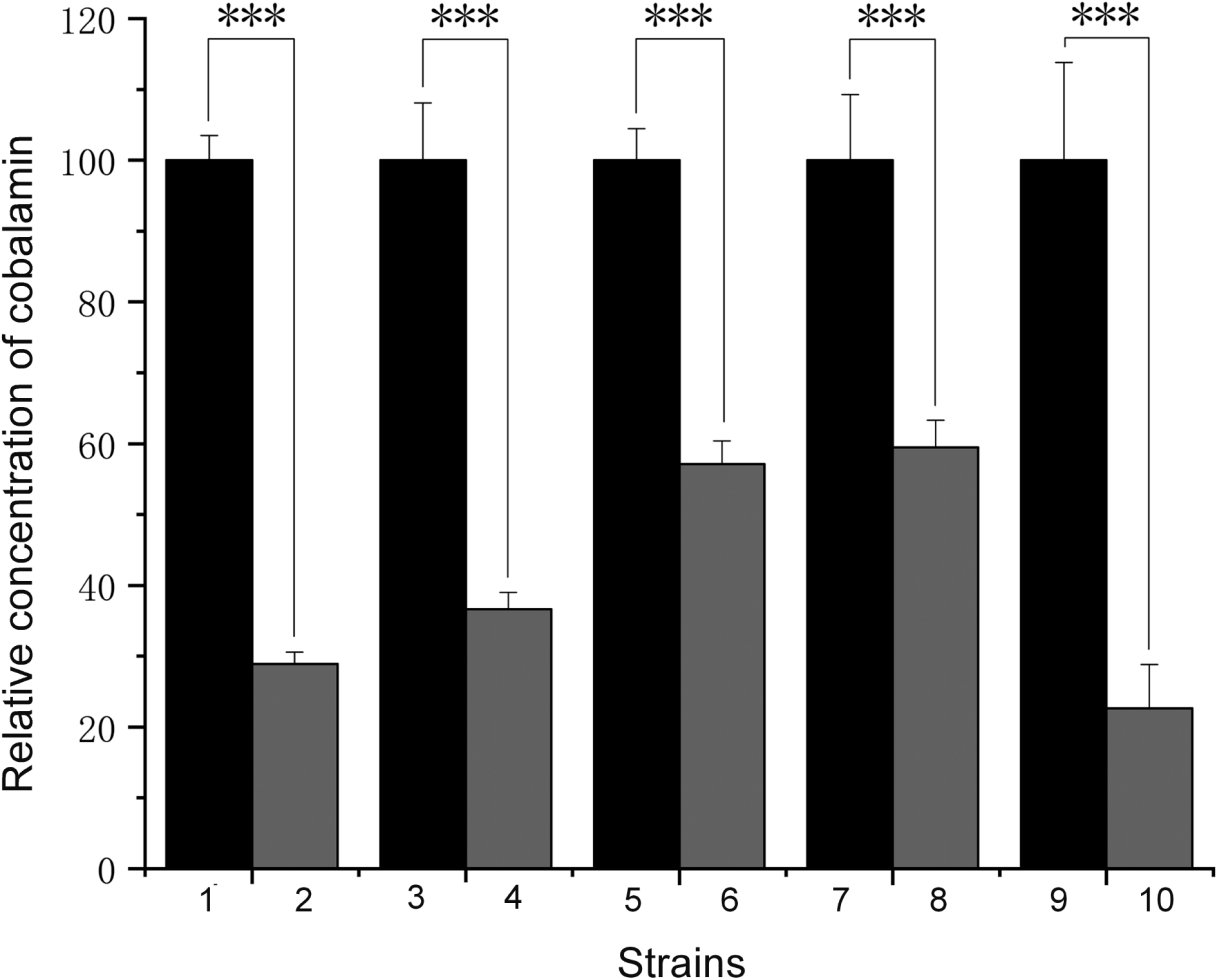
Expression of tRNA-Asp-AUC enhances biosynthesis of cobalamin. Isogenic pairs of strains, with (pSGTCG) and without (pSH152) the expressed tRNA-Asp-AUC, were assayed for cobalamin synthesis after 3 days growth in minimal medium. For each pair, the amount of cobalamin produced in the absence of the tRNA (even numbers) is indicated relative to the amount (100%) produced by the strain expressing the tRNA (odd numbers). 1 and 2: *S. coelicolor* M145; 3 and 4: *S. chattanoogensis*; 5 and 6: *S. filamentosus*; 7 and 8: *S. clavuligerus*; 9 and 10: *S. peucetius*. Error bars indicate SD, *** signifies *P* ≤ 0.001.

As reported above, proteome analysis indicated that the tRNA can lead to overexpression of cobalamin biosynthesis genes in *S. coelicolor*. Cobalamin is required for synthesis of S-adenosyl-I-methionine (SAM) and the latter is a methyl donating substrate implicated in synthesis of a wide range of antibiotics (51), including undecylprodiginines (52), the lipopeptides CDA and daptomycin (53), and daunorubicin (54). SAM can also indirectly activate actinorhodin biosynthesis (55). Consequently, we measured cobalamin synthesis in the five different *Streptomyces* species tested in this study. In each case, we measured significantly greater yields of cobalamin produced by the strains in which the tRNA was expressed (Figure 4).

## DISCUSSION

*Streptomyces* species typically encode an expanded range of distinct tRNA species compared to many other bacteria. For example, typical mesophilic species such as *S. coelicolor* encode 43 distinct tRNAs, whereas *E. coli* and *Bacillus subtilis* encode 40 and 32 tRNA species, respectively. Consequently, the requirement for decoding certain codons via non-canonical wobble base-pairing in *Streptomyces* is reduced. In this study we identify an additional species, tRNA-Asp-AUC, present in 0.7% of the *Streptomyces* sequence libraries deposited at NCBI. This tRNA is not found in other bacterial genera and indeed only five other bacterial species are predicted to encode non-homologous tRNA-Asp-AUC species.

The tRNA-Asp-AUC, which is constitutively expressed, clearly contributes to the efficiency of decoding GAU codons, as indicated by key experiments examining expression of modified GFP genes containing one or more GAT codons. Indeed, the unusual anticodon stem–loop, with two predicted unpaired base-pairs in the stem, is likely to affect interaction with the ribosome, and could influence translocation efficiency and accuracy (56). We cannot exclude that it may also decode GAC aspartate codons, but the presence of two copies of tRNA-Asp-GUC in the genomes of both SPC6 and *S. collinus* suggests this is unlikely or inefficient. In *S. coelicolor*, inclusion of one or more 5′ GAU codon reduces translation efficiency of eGFP mRNA unless the tRNA-Asp-AUC is co-expressed. In the absence of the co-expressed tRNA, translation of the mRNA with 6 GAU codons was only detected by microscopy in the reproductive hyphae of older cultures. We assume that expression of this eGFP in the aerial hyphae is dependent on inefficient wobble base-pairing involving the only aspartate tRNA, tRNA-Asp-GUC; as in other streptomycetes, the *S. coelicolor* genome contains two such tRNA genes of identical sequence. There is no evidence for Q tRNA modification of this tRNA-Asp-GUC, or other tRNAs with GUN anticodon sequences, in *Streptomyces*. In many bacteria, G34-to-Q34 modification can be synthesized *de novo* through a complex biosynthetic pathway, whereas other bacterial and eukaryotic species can salvage the Q nucleobase precursor queuine from queuosine-synthesising bacteria (57). We are not aware of any biochemical evidence for the Q biosynthetic pathway in *Streptomyces*: there are no homologs of genes encoding key pathway enzymes. In addition, the gene encoding the crucial tRNA guanine transglycosylase enzyme, responsible for insertion of Q into tRNA, is absent in streptomycete genomes, ruling out Q tRNA modification via a salvage pathway. In the absence of any evidence for this modification, inefficient wobble base-pairing is likely an evolved strategy to regulate the expression of genes containing one or more GAT codons. In researching the background to this, it is evident to us that surprisingly little is known about tRNA modification in *Streptomyces*, and only thiomethylation at position 37 in the anticodon loop has been reported, albeit with an important role on translation efficiency and consequently morphological differentiation and antibiotic production (58,59).

Translational regulation dependent on tRNA function in streptomycetes is precedented, in that the abundance of tRNA-Leu-UAA is known to determine translational efficiency of genes containing the very rare TTA codon (41). However, our study indicates a new paradigm, in that translation efficiency dependent on non-canonical base-pairing involving more frequent GAT codons, 48 times more abundant than TTA codons, is also important in regulating gene expression and impacts antibiotic production. Proteomic analysis of *S. coelicolor* revealed a range of proteins with different functions, whose overexpression was dependent on the novel tRNA, including antibiotic biosynthetic genes. Not all of these genes had an increased frequency of the GAT codon compared to the genome average, indicating a possible indirect relation between translation dependent on the tRNA and gene expression. Indeed, in relation to antibiotic biosynthesis, our analyses indicate that both pleiotropic and pathway-specific regulatory genes contain a much higher GAT codon content compared to the genome average.

We found that expressing the tRNA in *S. coelicolor* M145 resulted in precocious synthesis of four different antibiotics when the strain was grown on different growth media. Of note was synthesis of calcium-dependent antibiotic and coelimycin during growth on media that do not support production of inhibitory concentrations of either antibiotic by the non-recombinant strain. The observed increase in synthesis of undecylprodiginines and coelimycin correlated with increased expression of several biosynthetic enzymes detected in proteomic analyses. The tRNA also promoted increased synthesis of antibiotics by all other species we tested. This can be rationalised in part in terms of increased expression of pathway-specific and pleiotropic regulatory genes, and also increased co-factor synthesis. SAM, derived from cobalamin, is an important co-factor involved in antibiotic biosynthesis (51) and a significant increase in cobalamin synthesis dependent on the tRNA can thus impact antibiotic yields.

In conclusion, we have identified and demonstrated the function of a novel bacterial tRNA. Of note is that expression of this tRNA negates a requirement for less efficient translation dependent on wobble base-pairing in streptomycetes, leading to increased yields of all antibiotics tested, and establishes a new paradigm for the regulation of gene expression and antibiotic biosynthesis. Our data indicate that manipulation of translation efficiency in streptomycetes in this manner can not only improve yields of known antibiotics, but also permit expression in detectable yields of a cryptic antibiotic in *S. coelicolor*. The pleiotropic effects of enhancing translation efficiency in this way indicate that this is a rational and relatively simple generic strategy to incorporate in future antibiotic discovery programmes.

## DATA AVAILABILITY

All data in this paper is presented as figures, tables and in accompanying supplementary tables.

## Supplementary Material

gkac502_Supplemental_File
